# The Structure of the Talin Head Reveals a Novel Extended Conformation of the FERM Domain

**DOI:** 10.1016/j.str.2010.07.011

**Published:** 2010-10-13

**Authors:** Paul R. Elliott, Benjamin T. Goult, Petra M. Kopp, Neil Bate, J. Günter Grossmann, Gordon C.K. Roberts, David R. Critchley, Igor L. Barsukov

**Affiliations:** 1 School of Biological Sciences, University of Liverpool, Crown Street, Liverpool, L69 7ZB, UK; 2 Department of Biochemistry, University of Leicester, Henry Wellcome Building, Lancaster Road, Leicester, LE1 9HN, UK

## Abstract

FERM domains are found in a diverse superfamily of signaling and adaptor proteins at membrane interfaces. They typically consist of three separately folded domains (F1, F2, F3) in a compact cloverleaf structure. The crystal structure of the N-terminal head of the integrin-associated cytoskeletal protein talin reported here reveals a novel FERM domain with a linear domain arrangement, plus an additional domain F0 packed against F1. While F3 binds β-integrin tails, basic residues in F1 and F2 are required for membrane association and for integrin activation. We show that these same residues are also required for cell spreading and focal adhesion assembly in cells. We suggest that the extended conformation of the talin head allows simultaneous binding to integrins via F3 and to PtdIns(4,5)P2-enriched microdomains via basic residues distributed along one surface of the talin head, and that these multiple interactions are required to stabilize integrins in the activated state.

## Graphical abstract

**Graphical Abstract:**

**Figure:**
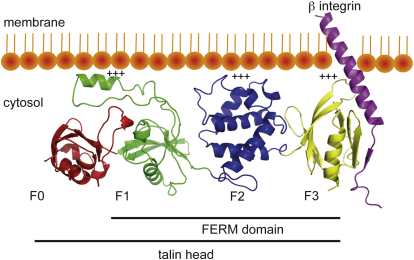

**Highlights:**

► Talin FERM domain has a novel open conformation ► Membrane association sites are distributed along one surface of the FERM domain ► These are critical for integrin activation and focal adhesion assembly ► Simultaneous integrin and membrane binding is favored by the linear talin structure

## Introduction

Proteins containing FERM domains are essential for a wide range of biological processes, including cell adhesion, motility, proliferation, and differentiation (Fehon et al., 2010). FERM domains have been shown to support numerous protein-protein and protein-lipid interactions and have therefore been the subject of intensive structural studies. A consensus FERM domain structure has emerged, largely from studies on the ezrin, radixin, moesin (ERM) family of proteins (Hamada et al., 2000; Pearson et al., 2000; Smith et al., 2003), which is characterized by a globular cloverleaf arrangement of three independently folded domains F1-3 (Pearson et al., 2000). This structure is also found in more distant ERM homologs such as focal adhesion kinase and band 4.1 protein (Ceccarelli et al., 2006; Han et al., 2000) and had been suggested as a prototype for all FERM domains (Pearson et al., 2000).

The FERM domain protein talin (∼270 kDa) plays a key role in activation of the integrin family of cell adhesion receptors and also provides a direct link between the cytoplasmic tail of the β-integrin subunit and the actin cytoskeleton. Talin consists of a ∼50 kDa N-terminal head and an elongated 220 kDa rod composed of amphipathic helical bundles (Critchley, 2009; Roberts and Critchley, 2009). The talin head contains a FERM domain which is similar to that found in ERM proteins, although it has two novel features: the F1 domain includes a 30 residue unstructured loop and is preceded by an additional domain (F0) which, like F1, has a ubiquitin-like fold (Goult et al., 2010). The integrin binding site is located in the F3 domain and, although a second integrin binding site exists within the talin rod (Gingras et al., 2009), only constructs containing the F3 domain are able to activate integrins (Calderwood et al., 2002). Interestingly, the kindlin family of FERM domain proteins has a similar domain structure to the talin head (Goult et al., 2009b) and kindlins synergize with talin in integrin activation (Moser et al., 2009). NMR and crystallographic studies show that there is an extensive binding interface between the talin F3 domain and the membrane proximal NPxY motif and helical region of β-integrin tails, and binding is thought to disrupt the salt bridge between the α- and β-integrin tails that normally keeps integrins in the low affinity state (Anthis et al., 2009; Garcla-Alvarez et al., 2003; Wegener et al., 2007).

In addition to the integrin binding site in F3, other regions of the talin head that are not directly involved in integrin binding are also important in integrin activation (Bouaouina et al., 2008). Thus, both the F2 and F3 domains contain groups of positively charged residues that contribute to integrin activation (Anthis et al., 2009; Wegener et al., 2007) and integrin clustering (Saltel et al., 2009) through interaction with negatively charged membrane phospholipids (Anthis et al., 2009; Saltel et al., 2009). Similarly, positively charged residues in the F1 loop that bind to negatively charged membranes are also required for integrin activation (Goult et al., 2010). Interestingly, the F1 loop forms a transient helix with positively charged residues aligned down one face, and this helix is stabilized by acidic phospholipids. We have proposed that folding of the loop brings the F1 domain closer to the membrane, and in this way contributes to integrin activation (Goult et al., 2010). The F0 domain is also essential for activation of β1-integrins and enhances the activation of β3-integrin (Bouaouina et al., 2008), although the mechanism is currently unclear.

The unique features of the talin head, such as the additional F0 domain which forms an intimate contact with F1 (Goult et al., 2010), and the large F1 loop indicate that the talin head has unique characteristics within the FERM superfamily. Indirect support for this comes from limited proteolysis experiments on the talin head which liberated a stable F2F3 fragment (Garcla-Alvarez et al., 2003). This is difficult to rationalize on the basis of the cloverleaf FERM domain structure in which the linker between F1 and F2 is buried in the hydrophobic core formed by the domain interfaces (Hamada et al., 2000). Here, we report the structure of the talin head, show that it adopts a novel open conformation, and identify the key elements that determine this alternative arrangement of the FERM subdomains. We propose that the extended conformation of the talin head allows for the simultaneous engagement of the multiple acidic phospholipid binding sites in the talin head with the membrane in a manner that stabilizes the integrin activation complex.

## Results

### Crystal Structure of the Talin Head Domain

**Figure 1:**
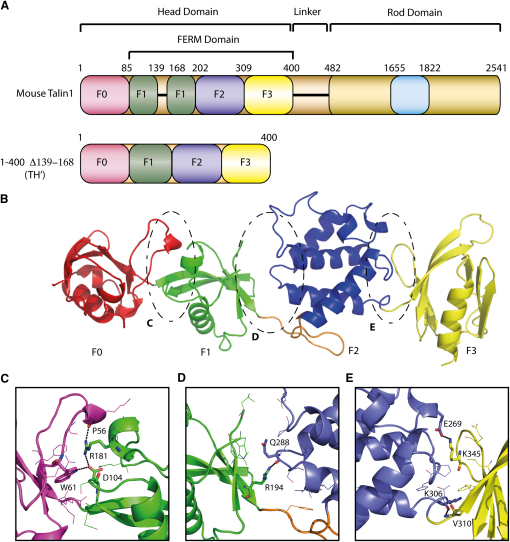
Domain Composition and Crystal Structure of the Talin1 Head Region (A) Overall domain architecture of talin1 and the fragment TH′ used for crystallization (residues 1–400 with F1 loop 139–168 removed). The autoinhibition domain 1655–1822 in the talin rod is marked in cyan. The color scheme for the talin domains is maintained in all figures. (B) Ribbon diagram of the structure of the talin1 head domain containing ubiquitin-like domains F0 (magenta) and F1 (green), acyl-CoA-binding protein-like domain F2 (blue) and PTB-like domain F3 (yellow). The F1-F2 linker region is marked in orange. The interfaces between the domains are encircled and expanded below. (C–E) Expanded regions of the interfaces between the F0-F1 (C), F1-F2 (D), and F2-F3 (E) domains showing the contacting residues. See also Figure S1.

Our early attempts to crystallize the talin FERM domain F1F2F3 (residues 86–400) were unsuccessful, probably because the F1 domain contains a large unstructured loop of approximately 30 residues that does not interact with other regions of the FERM domain (Barsukov et al., 2003). Recently, we determined the solution structure of the F1 domain alone and showed that the loop (residues 139–168) can be removed without altering the structure of F1 (Goult et al., 2010). We also established that the talin head contains an additional globular domain F0 at the N terminus that is closely associated with the F1 domain (Goult et al., 2010). Based on these results, we extended the FERM domain construct to include F0 (residues 1–400) and removed the unstructured F1 loop (residues 139–168), as illustrated in Figure 1A. The resulting talin polypeptide (residues 1–400 Δ139–168) denoted as TH′, crystallized, and the structure was determined by molecular replacement using the NMR structure of F0F1 (2KMA) and the X-ray structure of F2F3 (1MIX). The structure was refined to 1.9 Å (data collection and refinement statistics in Table 1), and the final R factor of 19.2% and R_free_ of 25.7% were obtained after the use of translational libration screw (TLS) translation restraints during the later stages of refinement. Overall, the model contains 358 residues and 561 solvent molecules with an average B factor of 18 Å^2^ (overall) and 25 Å^2^, respectively. One molecule was observed within the asymmetric unit and is shown in Figure 1B.

The structure of TH′ consists of four domains arranged in a novel linear configuration. Both F0 and F1 domains have ubiquitin-like folds, the F2 domain contains a core 4-helix bundle equivalent to that found in acyl-CoA-binding protein, and the F3 domain has a phosphotyrosine binding domain (PTB) fold. The previously determined structures of the F0F1 (2KMA) (Goult et al., 2010) and F2F3 (1MIX) (Garcla-Alvarez et al., 2003) double domains superimpose well onto the equivalent regions in the TH′ crystal structure with RMSDs of 2.09 and 1.09 Å, respectively. The interfaces F0-F1 and F2-F3 each form a network of close contacts (Figures 1C–1E) that stabilize the relative domain orientations. The F0-F1 interface, with a buried surface area of 860 Å^2^, includes hydrogen-bond interactions between the guanidinium group of R181 (F1) and the carbonyl of P56 (F0), as well as the carbonyl of D104 (F1) and the indole nitrogen of W61 (F0) (Figure 1C). These interactions supplement hydrophobic interactions centered around the aromatic ring of W61. Similarly, the F2-F3 interface, with a buried surface area of 650 Å^2^, includes a charge-charge interaction between the amino group of K345 (F3) and the carboxylate of E269 (F2), hydrogen bonds between the amide of V310 (F3) and carbonyl of K306 (F2), and a number of hydrophobic contacts (Figure 1E). In contrast, despite a similar buried surface area of 620 Å^2^, the F1-F2 interface is loosely packed with no specific interactions apart from a single hydrogen bond between the carbonyl of Q288 and the guanidinium group of R194 (Figure 1D).

### Small Angle X-Ray Scattering Shows an Extended Talin Head Structure in Solution

**Figure 2:**
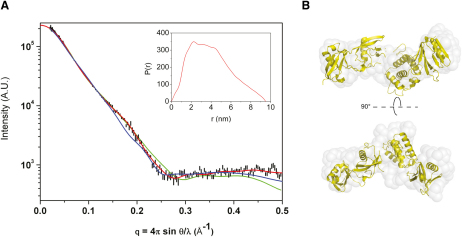
SAXS Analysis of the Talin1 Head (A) Comparison between the experimental scattering profile (black) and profiles calculated from the crystal structure (blue), from ab initio shape reconstruction with GASBOR (red) and by the BUNCH method (green), generated with the two double domains F0F1 and F2F3 as independent rigid bodies. The error bars of the experimental curve represent 1 SD. The goodness of fit of the crystal structure, GASBOR, and BUNCH profiles versus experimental data is χ^2^ = 3.8, 1.6, and 3.2, respectively. Inset shows the pair distance distribution function *P(r)* for the talin head at 4°C. (B) GASBOR shape envelope (transparent gray surface) superimposed with the BUNCH model (yellow). The two orientations shown are related by a 90° rotation around the horizontal axis. See also Figure S2.

The conformation of TH′ in solution was analyzed by small angle X-ray scattering (SAXS). Calculation of the theoretical scattering profile from the crystal structure showed a significant deviation from the experimental curve (Figure 2A), indicating that the structure in solution has some differences relative to the crystal form. Ab initio bead modeling with GASBOR (Svergun et al., 2001) resulted in a scattering profile with a substantially improved goodness of fit value compared with that obtained from the crystal structure (χ^2^ = 1.6 and 3.8, respectively), and no systematic deviations from the experimental scattering profile (Figure 2A). The GASBOR envelope indicates an extended conformation, although it is not as linear as in the crystal structure. It can be approximated by two elongated shapes joined end-to-end at an angle of ∼120°, with the size of each shape close to that of a double domain in the TH′ structure (Figure 2B). Reconstruction with BUNCH (Petoukhov and Svergun, 2005) using F0F1 and F2F3 as two rigid bodies fits well within the GASBOR shape envelope (Figure 2B), although the calculated scattering profile has some systematic deviations from the experimental data (Figure 2A). The fit is improved when a mixture of conformation is allowed in the Ensemble Optimization Method (EOM) (Bernado et al., 2007). All the structures obtained by the EOM analysis have an open domain arrangement, while they differ by the angle between the double domains (see Figures S2A and S2B available online). Overall, the SAXS analysis is consistent with an open extended conformation of the talin head in solution and suggests a degree of flexibility at the F1-F2 interface that correlates with the absence of specific contacts at this interface.

SAXS analysis demonstrates similarity between the structure in solution and in the crystal for TH′ lacking the F1 insert loop. This loop is unstructured in the F0F1 fragment, and its removal does not induce any structural changes in that fragment (Goult et al., 2010). As the loop also remains unstructured in the F1F2F3 fragment (Barsukov et al., 2003), its removal in TH′ is expected to preserve the structure of the full talin head. This conclusion is supported by the comparison of the [^1^H,^15^N]-HSQC spectra of TH′ and the full talin head (residues 1–405) in Figure S2C. Despite the large molecular weight of the proteins, the spectra have sufficient quality to demonstrate that the removal of the loop leads primarily to the disappearance of intense sharp signals in the spectra of TH′ that correspond to unstructured flexible regions, while the broad resonances from the folded domains remain virtually unchanged.

### The Noncanonical Arrangement of Domains within the Talin Head

**Figure 3:**
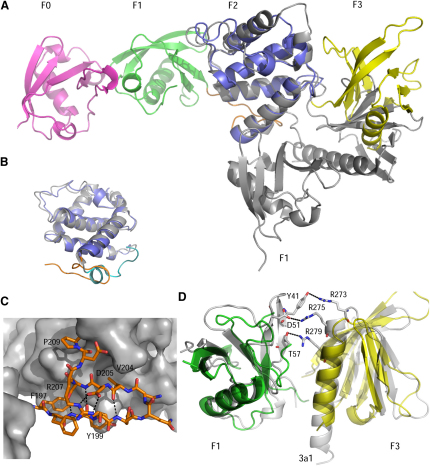
Structural Features Specific to the Talin1 FERM Domain (A) Comparison of the talin head structure and the structure of the radixin FERM domain (gray) based on the superposition of the F2 domain of each protein. (B) Superposition of the F2 domain of talin (blue) and radixin (gray) showing different orientations and conformations of the F1-F2 linker region. Talin linker region is marked in orange, radixin in cyan. (C) Docking of the β-hairpin formed in the F1-F2 linker region of talin (stick representations) against the surface of the F2 domain (gray). Residues stabilizing the hairpin packing are marked. The hydrogen bonds in the hairpin are represented by dashed lines. The molecule orientation is as in (B). (D) Modeling of the F1 and F3 domains of talin (green and yellow, respectively) onto the radixin structure (PDB ID 1GC7; gray). Residues forming contacts between the F1 and F3 domains of radixin are shown in stick representation and marked. No similar contacts can be formed in talin due to residue deletions and substitutions. See also Figure S3.

All FERM domain structures reported to date have a compact cloverleaf domain arrangement that is dramatically different to the open structure of the talin head. Within the ERM domain family, radixin has the highest sequence similarity to TH′ (26% identity). Although individual domains of talin superimpose well with the equivalent regions of radixin (PDB ID 1GC7), the overall domain arrangement in the two proteins is quite different. Superposition of the two structures on the F2 domain (Figure 3A) reveals that talin F3 rotates by ∼30° from the position of F3 in radixin, as previously reported for the isolated F2F3 structure (Garcla-Alvarez et al., 2003). The talin F1 domain rotates around F2 by ∼90° in the opposite direction, positioning F1 and F3 on the opposite sides of F2 in a linear arrangement. The F1-F2 interface in radixin is extensive (buried surface area of 1540 Å^2^) while these domains are more loosely packed in TH′ (buried surface area of 620 Å^2^). Inspection of the TH′ crystal packing demonstrates that no symmetry-related molecules exhibit a canonical FERM cloverleaf domain arrangement through a domain-swap mechanism (Figure S3A).

The key areas that stabilize the cloverleaf conformation of the radixin FERM domain are the F1-F2 linker region and specific contacts between the F1 and F3 domains (Hamada et al., 2000). Despite the overall sequence similarity between radixin and talin, these particular regions show large sequence and structure variations that can be correlated with the different FERM domain conformations. The 13 residue long linker that connects F1 and F2 in TH′ contains a highly conserved in ERM family and band 4.1 motif KF(F/Y) (Garcla-Alvarez et al., 2003) at the N terminus, but shows large difference to these proteins in the rest of the sequence (Figure S1). In radixin, the linker is packed against F2 through hydrophobic contacts involving side chains of P86, V89, and L93 (Figures S4C and S4D). The packing is facilitated by the formation of a helical turn that positions the side chains of V89 and L93 next to each other. The orientation of the F2-F3 linker positions F1 in close proximity to F3. The three domains enclose the conserved KF(F/Y) motif inside a hydrophobic core that is stabilized by the aromatic ring contacts.

The F1-F2 linker region in talin (residues 196–208) adopts a distinctly different β-hairpin-like conformation in the F198-S205 region (Figures 3B and 3C; Figure S4B). The hairpin is anchored to the surface of the F2 domain through hydrophobic interactions involving the side chains of F198 and V204 at the opposite ends of the hairpin. Additionally, a close polar contact is formed between the amide side chain of N203 at the end of the β-hairpin and the guanidinium group of R303 in the 2α4 helix of F2. The packing of the hairpin against the F2 surface is facilitated by the turn in the R207-D205 region stabilized by the salt bridge between the side chains of R207 and D205. The orientation of the turn relative to the helix 2α1 is determined by P209 that naturally changes the direction of the peptide chain. The packing of the linker region against F2 positions the F1 domain on the opposite surface of F2 relative to F3. Interestingly, only F198 of the conserved KF(F/Y) motif plays a structural role in talin, while the other two residues do not make any distinct contacts.

The conformation of the F1-F2 linker in TH′ is well defined, as evidenced by the electron density (Figure S4A), and no distinct crystal packing contacts are observed (Figure S3C), suggesting the same linker conformation would be maintained in solution. Limited proteolysis experiments using trypsin resulted in a proteolytically resistant fragment 196–400 (Garcla-Alvarez et al., 2003) that places the cleavage site to the N terminus of the linker. The resistance to proteolysis suggests that the linker is well structured in solution while the preceding residues at the C terminus of F1 are accessible, in agreement with the linear structure of TH′ and the flexibility in the F1-F2 connection. In the cloverleaf conformation found in other FERM domains, both the linker region and the C terminus of F1 are buried in the middle of the structure (Hamada et al., 2000).

The contact between the F1 and F3 domains in radixin is mediated through the positively charged side chains of R273, R275, and R279 in the N-terminal part of the helix 3α1 (F3) that form a range of ionic and hydrogen bond interactions with residues in the 1β3-1β4 loop (F1), as illustrated in Figure 3D (Hamada et al., 2000). Although F1 and F3 are distant in the talin head, we modeled a closed cloverleaf conformation of TH′ using the radixin structure. In talin, the 3α1 helix is shortened due to a 3 residue deletion (Figure 3D; Figure S1), which removes residues equivalent to R273 and R275. The shortening of the helix reduces the contact area and abolishes the stabilizing interactions in the modeled cloverleaf talin conformation. Additionally, R279 is replaced by the negatively charged E262, further reducing potential favorable contacts. Although the structure of talin F3 has been reported previously (Garcla-Alvarez et al., 2003), the significance of these differences only becomes clear now that we have determined the full talin head structure. Interestingly, in the distant homolog FAK, these positively charged residues are also absent (Ceccarelli et al., 2006). However, the absence of the charge-charge interaction in FAK is compensated by a small rotation of F3 relative to the other domains, leading to the formation of alternative stabilizing contacts. Additionally, the linker between the FERM domain and the kinase domain in FAK sits across the F1-F3 interface, further stabilizing the closed FERM domain conformation (Ceccarelli et al., 2006). Sequence and structure comparison demonstrates that equivalent contacts are absent in the corresponding model of the talin head.

Note that the above comparison only demonstrates that no stabilizing contacts between talin F1 and F3 can be formed in the relative domain orientations characteristic to ERM proteins or FAK, and does not exclude the possibility of a favorable interaction with significantly different relative orientation of F1 and F3. In addition, a “closed” form of the TH may be stabilized by interactions with the talin rod in the autoinhibited form of the molecule, or in talin complexes with other domains or proteins. If true, this would open the intriguing possibility of functional regulation of TH through large-scale domain rearrangement. The large size of the F1-F2 linker allows for repositioning of F1. In summary, the detailed structure analysis indicates that the contacts of F1-F2 linker region and the surface properties of the F3 domain favor a noncanonical linear conformation of the talin head.

### Charge Distribution in the Talin Head and Interaction with Negatively Charged Phospholipids

**Figure 4:**
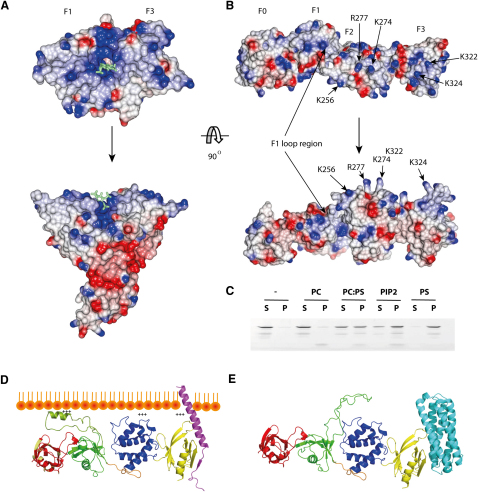
Distribution of Functional Residues on the Surface of the Talin1 Head (A and B) Comparison of the surface charge distribution in radixin (1GC7) (A) and TH′ (B). Both proteins have one face that is predominantly positively charged (top). For TH′, the positive charges are distributed along the entire molecule, while in radixin the positive charges are clustered around the F1-F3 interface. The molecules are additionally shown rotated −90° along the x axis. Residues in talin identified as being involved in interactions with the phospholipid bilayer (Anthis et al., 2009; Wegener et al., 2007) are indicated. Their location on a single face suggests that the top view is the membrane-binding surface in talin. The inositol-3-phosphate binding site on radixin (shown as a light green stick structure) identifies the membrane-binding surface in radixin. The positively charged residues of radixin involved in membrane binding are not conserved in talin1. (C) TH′ association with negatively charged phospholipids demonstrated by pull-down of TH′ with large multilamellar vesicles. Lanes 1, 2, no lipid; lanes 3, 4, POPC; lanes 5, 6, 1:4 POPS:POPC; lanes 7, 8, 1:19 PIP2:POPC; lanes 9, 10, POPS. S, supernatant; P, pellet. (D) Model of complex between talin head and the β3-integrin cytoplasmic domain based upon the integrin/talin F3 structure (Wegener et al., 2007) docked against the membrane surface. The helical structure in the F1 insert loop was modeled following (Goult et al., 2010). In this orientation of TH′ all membrane interacting residues are facing the membrane and indicated by plus signs. The linear arrangement of domains in the talin head allows all membrane-binding sites to engage simultaneously in an orientation optimal for the integrin activation. Conserved patch on F0 is marked in light orange. (E) Autoinhibited complex between the talin head and the rod domain 1655–1822 based on the F3/1655–1822 model of (Goult et al., 2009a). The F1 insert loop, absent in TH′, was modeled using the NMR structure of the F1 domain (Goult et al., 2010). See also Figure S4.

A range of FERM domain proteins bind to membrane inositol phospholipids through a highly positively charged surface at the F1-F3 interface (Figure 4A) (Barret et al., 2000; Hamada et al., 2000). In the extended structure of the talin FERM domain, F1 and F3 are spatially separated, and the majority of the residues equivalent to the lipid binding patch in ERM proteins are either absent or do not have a positive charge (Figure S1). In addition, no extensive positively charged patch exists on the surface of the talin head, although one face of the molecule is predominantly positively charged (Figure 4B; Figure S1). Despite the lack of an extensive positively charged cluster, TH′ retains the ability to bind to negatively charged phospholipid bilayers as shown using a cosedimentation assay (Figure 4C). No association was detected between TH′ and uncharged multilamellar vesicles (MLVs) containing POPC. Incorporation of 20% POPS resulted in an equal distribution of TH′ between the pellet and the supernatant, while the presence of 5% PtdIns(4,5)P_2_ (PIP_2_) led to the majority of TH′ in the pellet. In the presence of pure POPS MLVs, all TH′ was associated with the vesicles. These results are consistent with the reported interaction of the ∼50 kDa talin head fragment (Niggli et al., 1994) and of individual talin head domains (Anthis et al., 2009; Goult et al., 2010) with negatively charged vesicles. The interaction is dependent on the negative charge rather than the composition of the membrane, as shown by the similar effect of POPS and PIP_2_. The affinity of binding is affected by the charge density, and is higher for PIP_2_ than POPS due to the presence of two negatively charged phosphate groups in the PIP_2_ lipid head group, as evidenced by the lower PIP_2_ than POPS concentration required to achieve a similar pull-down level (Figure 4C).

All residues in the talin head identified as important for membrane association are located on the predominantly positively charged surface (Figure 4B). They include K256, K272, K274, and R277 in F2 (Anthis et al., 2009; Garcla-Alvarez et al., 2003), as well as K322 (Wegener et al., 2007) and K324 (Saltel et al., 2009) in F3. In addition, we have recently shown that the F1 loop interacts with negatively charged membranes through formation of a helical structure with a high concentration of positive charges on one face (Goult et al., 2010). In solution, the helix is transient and is stabilized by the presence of negatively charged membranes. The F1 loop is inserted between strands β3 and β4 which positions it at the same surface as other membrane-interacting residues in F2 and F3. The location of all the membrane-binding residues on the same face of the talin head indicates that they can all simultaneously associate with the membrane, thus generating high affinity binding through multiple dispersed interactions (Figure 4D).

### Basic Residues in the F1 and F2 Domains Are Important in Talin-Mediated Cell Spreading and FA Assembly

**Figure 5:**
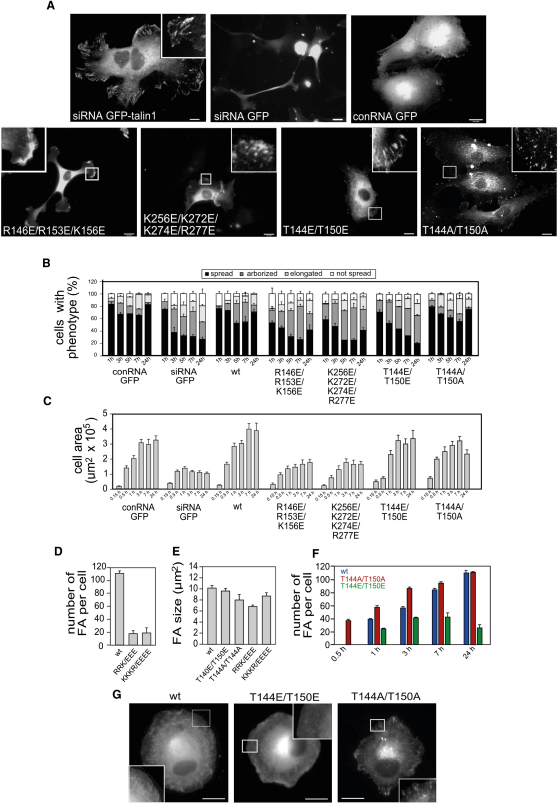
Mutations in Basic Residues in the Talin1 F1 and F2 Domains Inhibit Cell Spreading and FA Formation HUVECs were transfected with a talin1 siRNA (or a control RNA; conRNA) plus constructs encoding either wild-type GFP-talin1, the GFP-talin1 FERM domain mutants indicated, or GFP alone. Cells were replated on glass coverslips 72 hr posttransfection and then fixed/stained and imaged 24 hr later. (A) Epifluorescence images showing the localization of GFP or GFP-talin1. (B) Quantitative analysis of cell morphology at various time points after replating (expressed as mean ± SEM) assessed by the following criteria: “spread” = area of cytoplasm is three times bigger than the area of the nucleus; “arborized” = cell with more than five prominent protrusions or more than three axes; “elongated” = cell length at least five times bigger than cell width; “not spread” = area of cytoplasm is equal to or less than three times the area of the nucleus. (C) Time course of cell spreading based on cell area. (D and E) Number (D) and size (E) of GFP-talin1-positive FA quantified using ImageJ 24 hr after replating. (F) Time course of FA formation. Number of GFP-talin1-positive FA per cell is expressed as mean ± SEM. (G) Epifluorescence images of cells treated as described above 30 min after replating, showing the localization of the GFP-talin1 constructs indicated. Wild-type (wt) GFP-talin1. Scale bars, 10 μm.

Although the talin F3 domain is solely responsible for binding β-integrin tails and is essential for integrin activation, basic residues in F1 and F2 that bind acidic phospholipids are also essential for integrin activation (Anthis et al., 2009; Goult et al., 2010). We therefore sought to evaluate the importance of these basic residues in a cellular context. Studies on talin function in cells are complicated by the fact that most cell types express two closely related talin isoforms, and depletion of talin1 leads to the rapid compensatory upregulation of talin2 (Zhang et al., 2008). However, we have recently found that human umbilical vein endothelial cells (HUVECs) only express talin1, and siRNA-mediated knockdown of talin1 results in a marked reduction in cell spreading and focal adhesion (FA) assembly, a phenotype that can be rescued by expression of full-length GFP-tagged mouse talin1, but not GFP alone (Figure 5) (Kopp et al., 2010). Significantly, neither GFP-talin1 containing a R146E/R153E/K156E mutation in the F1 loop nor a GFP-talin1 F2 mutant (K256E/K272E/K274E/R277E) was able to rescue the talin1 knockdown phenotype, and after 24 hr, only ∼42% of cells were spread, with a high percentage of cells remaining arborized and elongated (Figures 5A and 5B). The spread cell area was also significantly reduced compared with cells expressing wild-type talin1 (Figure 5C). Both mutants failed to support FA assembly in the majority of cells (Figure 5A), and there was a marked reduction in the number though not size of FA (Figures 5D and 5E). As these mutations do not perturb the folding of the F2 domain (Anthis et al., 2009), the biological effect is directly associated with the charge reversal. The results clearly demonstrate that the basic regions on both the F1 and F2 domains play a key role in talin-dependent cell spreading and FA assembly.

The basic region in the F1 loop contains two of the most abundant phosphorylation sites in platelet talin, T144 and T150 (Ratnikov et al., 2005), and we have shown that substituting these residues for glutamates significantly reduced the ability of the talin1 head to activate β1-integrins expressed in CHO cells (Goult et al., 2010). In line with these observations, a GFP-talin1 T144E/T150E phosphomimetic mutant only partially rescued the talin1 knockdown phenotype (Figure 5A), and the cells were more arborized than controls (Figure 5B), although the spread cell area was similar to that of controls (Figure 5C). Time course experiments showed that FA assembly was delayed, and by 24 hr cells expressing the T144E/T150E mutant had far fewer GFP-positive FAs than cells expressing wild-type GFP-talin1 (Figure 5F), although FA size was not affected (Figure 5E). In contrast, although cells expressing the nonphosphorylatable T144A/T150A mutant spread to about the same extent as controls (Figure 5B), FA assembly was much more rapid than in cells expressing wild-type GFP-talin1, and >30% of the total number of FA were formed as early as 0.5 hr after plating (Figures 5F and 5G). The results suggest that phosphorylation of T144/T150 negatively regulates the activity of the talin head, and that dephosphorylation of these residues is required for rapid FA assembly.

## Discussion

The FERM domain has traditionally been viewed as a uniform superfold of three distinct domains stabilized in a globular cloverleaf arrangement. Our structure of the talin head demonstrates that this view is oversimplified and that the structural variation across the FERM domain family is likely to be larger than hitherto expected. It remains to be seen if talin is a unique outlier in this superfamily or the first of a number of proteins with noncanonical FERM domains. In this respect, the kindlin family is particularly interesting, as kindlin FERM domains are closer to talin in evolutionary terms than other FERM domain proteins (Bretscher et al., 2002), and share talin-specific features such as an N-terminal F0 domain and a large F1 loop (Goult et al., 2009b). Moreover, kindlins synergize with talin in integrin activation (Moser et al., 2009).

Comparison with other ERM proteins reveals that the open talin head structure correlates with sequence differences in the F1-F2 linker and the F3 domain. Deletions and substitutions in the 3α1 helix within talin F3 result in the loss of conserved positive charges which in ERM proteins form salt bridges with residues in the F1 domain that stabilize the cloverleaf conformation. The talin F1-F2 linker contains residues that facilitate the formation of a β-hairpin that stabilizes packing against the F2 domain, pulling F1 close to F2 and orienting it on the opposite face relative to the F3 domain, so that all three domains have a linear configuration. The interfaces between F0-F1 and F2-F3 include substantial numbers of specific contacts that fix the relative domain orientation, while the contacts between F1 and F2 are limited and nonspecific, and a degree of flexibility at the F1-F2 interface is indicated by the SAXS data. The NMR spectra demonstrate that the F1 loop in the full talin head remains dynamic and does not interact with the folded domains.

The talin head structure allows us to consolidate the observations reported for individual talin domains into a single model. In inactive autoinhibited talin, the F3 domain in the talin head binds to a helical bundle in the talin rod (residues 1655–1822) (Goult et al., 2009a), and this masks the integrin binding site in F3 (Goult et al., 2009a; Goksoy et al., 2008). Modeling the complex between F3 and the rod domain (1655–1822) (Goult et al., 2009a) onto the structure of the talin head shows that the rod domain would also disrupt the F2F3 interaction with the membrane through a steric clash with the membrane surface (Figure 4E). However, the position of the F1 domain, far removed from F3 in the linear talin head structure, means that the extended F1 loop remains fully available for binding to the membrane even in autoinhibited talin. We have proposed a so-called “fly-casting model” in which contact between the F1 loop and PIP_2_-rich membrane microdomains induces helix formation, and this in turn shortens the loop and pulls the talin head close to the membrane (Goult et al., 2010). The linear arrangement of the talin head domains is optimal for the subsequent engagement of basic residues in F2 and F3 with acidic membrane phospholipids (Figure 4D), and the flexibility at the F1-F2 interface may allow additional structural adjustments to fit the membrane surface. However, for this to happen, the interaction between F3 and the rod domain must be broken. Interestingly, this interaction involves binding of basic residues in the F3 integrin activation loop to acidic residues in the talin rod (Goult et al., 2009a), an interaction that can be inhibited by PIP_2_ (Goksoy et al., 2008). This suggests that the activation of talin may depend on the negative charge density at the membrane. When this is low, the interaction energy may not be sufficient to inhibit F3/rod binding, but an increase in negative charge density might displace the rod domain and activate talin, allowing engagement of F2F3 with the membrane and of F3 with β-integrin tails. Activation of talin by PIP_2_ has been demonstrated in vitro (Martel et al., 2001), and integrin-mediated adhesion of cells to fibronectin has been shown to induce a 2-fold increase in PIP_2_ concentration (McNamee et al., 1993). Moreover, the talin head binds PIP kinase type 1γ which has been shown to play a key role in FA assembly (Di Paolo et al., 2002; Ling et al., 2002). However, talin can also be activated by the Rap1/RIAM pathway (Han et al., 2006; Lee et al., 2009; Watanabe et al., 2008), and the relative contributions of this and PIP_2_ in talin activation remain to be explored.

The involvement of multiple binding interactions distributed along the entire length of the talin head in the assembly of activated talin-integrin complexes correlates with the mutational analysis of talin. Thus, we have recently reported that basic residues in both the F1 loop (Goult et al., 2010) and on F2 (Anthis et al., 2009) are required for integrin activation, but not binding, and we show here that these same basic residues are also essential for cell spreading and FA assembly in HUVEC. Similarly, basic residues on both F2 and F3 are required for talin head-mediated Mn^2+^-dependent αvβ3-integrin clustering (Saltel et al., 2009). We have also previously shown that substitution of the two major phosphorylation sites in talin (T144/T150 in the F1 loop) with glutamates inhibits the association of F1 with acidic phospholipids, and also integrin activation (Goult et al., 2010). The fact that substituting these residues with alanine led to acceleration of FA assembly in HUVEC strongly suggests that phosphorylation of T144/T150 negatively regulates talin function by inhibiting the association of F1 with the membrane.

Recent NMR (Wegener et al., 2007) and crystallographic (Anthis et al., 2009) studies show how engagement of talin F3 with the membrane proximal NPxY motif and helical region of β-integrin tails disrupts the salt bridge between the α- and β-integrin tails that keeps the integrin in the low affinity state. However, interaction between the transmembrane domains of the integrin subunits also helps to maintain integrins in the low affinity state (Lau et al., 2009). The crystal structure of β1D-integrin tails complexed to the F2F3 domains of talin2 (Anthis et al., 2009) shows that for the basic residues in F2 and F3 to bind to acidic membrane phospholipids, the talin head must rotate by ∼20°. Anthis et al. (2009) suggest that this rotation exerts force on the transmembrane domains and leads to their separation, thus contributing to integrin activation. The structure of the complete talin head is consistent with this proposal and suggests that the activated conformation of integrins is stabilized by multiple charge-charge interactions with acidic membrane phospholipids along the entire surface of the extended talin head. Intriguingly, the F0 domain, which is also required for efficient β1- and β3-integrin activation (Bouaouina et al., 2008), contains an extensive patch of conserved residues that is fully exposed in the active talin head (Figure 4D) (Goult et al., 2010). The position and the conservation of this patch suggest that additional interactions between F0 and membrane components may also contribute to the ability of talin to activate integrins.

## Experimental Procedures

### Protein Expression and Purification

Mouse talin1 head domain with the loop removed (residues 1–400 Δ139–168; TH′) was cloned into pET151/D (Invitrogen), which contains an N-terminal hexa-histidine tag followed by a TEV protease site. Recombinant protein was expressed in the *Escherichia coli* strain *Rosetta* (DE3) at 30°C in 2YT medium. Once the cell density had reached an OD_600_ of 0.6, the cells were induced overnight at 18°C with 0.2 mM IPTG. Recombinant TH′ was purified by immobilized metal-affinity chromatography (IMAC). The hexa-histidine tag was removed by the addition of TEV protease overnight followed by reverse IMAC before being further purified by cation exchange chromatography. The purified protein was judged to be 98% pure by SDS-PAGE analysis. The purified protein was concentrated to approximately 10 mg/ml prior to crystallization.

### Crystallization and Data Collection

Crystals of the TH′ were grown by sitting-drop vapor diffusion. In brief, 10 mg/ml of TH′ in 20 mM MES, 100 mM NaCl, 1 mM DTT (pH 6.8) was mixed with an equal volume of reservoir solution containing 100 mM Tris, 21% PEG 4,000, 200 mM (NH_4_)_2_SO_4_ (pH 7.4). Crystals appeared as multiple thin plates stacked on top of each other within 1 week at 4°C. Further optimization of the crystals using additives proved unsuccessful, although this was not essential as the crystals could be separated from one another. Prior to data collection, a single crystal was transferred into the reservoir solution with the addition of 30% ethylene glycol, and flash frozen in liquid nitrogen. Diffraction data were collected from a single crystal at the SLS (PSI, Villigen, Switzerland) at beamline X06SA. In total, 90 images were collected with 1° oscillations at a fixed wavelength of 0.725 Å on a Pilatus 6M detector with a crystal to detector distance of 550 mm. The data were processed and integrated with MOSFLM (Leslie, 1992) with the autoindexing solutions indicating a primitive orthorhombic cell. The data were initially scaled using SCALA (Evans, 2006) in *P*222 prior to molecular replacement.

### Structure Determination and Refinement

Molecular replacement using PHASER (McCoy et al., 2007) focused on sampling for each subdomain, domains F2F3 from the chick structure (1MIX), and F0F1 from NMR ensembles (2KMA) to sample alternative space groups. A valid molecular replacement solution was only found in space group *P*22_1_2_1_, with the crystallographic statistics shown in Table 1. Following molecular replacement with space group *P*22_1_2_1_, electron density corresponding to the linker regions between each subdomain allowed unambiguous model building using COOT (Emsley and Cowtan, 2004). Model refinement consisted of iterative restrained refinement using REFMAC5 (Murshudov et al., 1997) and COOT (Emsley and Cowtan, 2004). In the final cycles of refinement, translational libration screw (TLS) refinement was used (Painter and Merritt, 2006) whereby each subdomain was processed as an individual TLS group, determined by the TLS motion determination (TLSMD) server (Painter and Merritt, 2006). The use of TLS restraints improved the R-factor by 4% with a final R-factor of 19.2% and R_free_ of 25.7% (Table 1). Model validation was performed by PROCHECK (Laskowski et al., 1993) and the MOLPROBITY (Davis et al., 2007) server. The final model consisted of 358 residues and 561 solvent molecules. Ramachandran analysis showed 98.9% residues were in the favored region and 1.1% residues in the additionally favored regions.

### Small Angle X-Ray Scattering

SAXS experiments were carried out at station 2.1 of the Synchrotron Radiation Source at Daresbury. The date were analyzed using GASBOR (Svergun et al., 2001), BUNCH (Petoukhov and Svergun, 2005), and EOM (Bernado et al., 2007) software (see Supplemental Experimental Procedures for details).

### Phospholipid Cosedimentation Assay

Large multilamellar vesicles were prepared essentially as described previously (Anthis et al., 2009); see Supplemental Experimental Procedures for details.

### Cell Culture and Transfection

Human umbilical vein endothelial cells (HUVECs) were purchased from PromoCell at passage 2 and grown according to the manufacturer's instructions in Endothelial Cell Growth Medium 2 (PromoCell) at 37°C in 10% CO_2_. For siRNA knockdown of human talin1, Stealth Select RNAi (Invitrogen cat # 1299003; oligo 804 seq: CCAAGAACGGAAACCUGCCAGAGUU) was used. A Universal Stealth RNA siRNA was used as negative control (conRNA). Subconfluent cells were trypsinized, washed in PBS, and electroporated (6 × 10^6^ cells/ml) using a Microporator (Invitrogen) with 100 pmol of siRNA. Cells were grown on tissue culture plastic for 72 hr, and replated at a density of 4 × 10^4^ cells on 16 mm glass coverslips for image analysis a further 24 hr later. Where appropriate, cells were cotransfected with the full-length mouse talin1 cDNA cloned into pEGFP-N1 (Clontech) as described elsewhere (Kopp et al., 2010). Cells were fixed in 3.5% formaldehyde in PBS-ME (containing 3 mM MgCl_2_ and 3 mM EGTA) for 10 min at room temperature and mounted on glass slides. Images were taken with a 40× oil immersion objective on an inverted Nikon TE300 microscope equipped with a Hamamatsu ORCA-ER digital camera, and an X-cite 120 fluorescence illumination system controlled by Improvision's Openlab software. Note that the ability of GFP to dimerize could potentially change the properties of GFP-tagged talin1. siRNA depletion of talin1 in human endothelial cells caused defects in cell spreading and FA assembly. These were fully reversed by expression of GFP-tagged full-length mouse talin1 indicating that the GFP-tag had no obvious affect on the function of the expressed protein.

## Figures and Tables

**Table 1:** Data Collection and Refinement Statistics for the Talin Head Domain

	TH′, 1–400 Δ139–168
**Data collection**	
Space group	P2_1_2_1_2
Cell dimensions	
* a*, *b*, *c* (Å)	40.3, 72.2, 162.8
Resolution (Å)	72–1.9 (2.05–1.94)
R_merge_	10.9 (38.7)
*I* / σ*I*	8.3 (3.3)
Completeness (%)	96.7 (99.8)
Redundancy	2.9 (3.2)

**Refinement**	
Resolution (Å)	43–1.9
No. reflections	34521
R_work_ / R_free_	0.192/0.257
No. atoms	
Protein	2890
Water	561
B factors (Å^2^)	
Protein	18.5
Water	36.5
Rmsd	
Bond lengths (Å)	0.023
Bond angles (°)	1.840

## Accession Numbers

Atomic coordinates for talin 1-400 (Δ139-168) have been deposited in the Protein Data Bank under accession number 3IVF.

## Supplemental Information

Document S1. Four Figures, Supplemental Experimental Procedures, and Supplemental References
