# Supplementary material for: The Structure of the Talin Head Reveals a Novel Extended Conformation of the FERM Domain
Source: Structure. 2010 Oct 13;18(10-13):1289–99. doi: 10.1016/j.str.2010.07.011 (PMC2977851; doi:10.1016/j.str.2010.07.011)
Supplement: Document S1. Four Figures, Supplemental Experimental Procedures, and Supplemental References [file mmc1.pdf]

Supplemental Information

The Structure of the Talin Head Reveals a Novel Extended Conformation of the FERM Domain

Paul R. Elliott, Benjamin T. Goult, Petra M. Kopp, Neil Bate, J. Günter Grossmann, Gordon C. K. Roberts, David R. Critchley, and Igor L. Barsukov

Figure S1

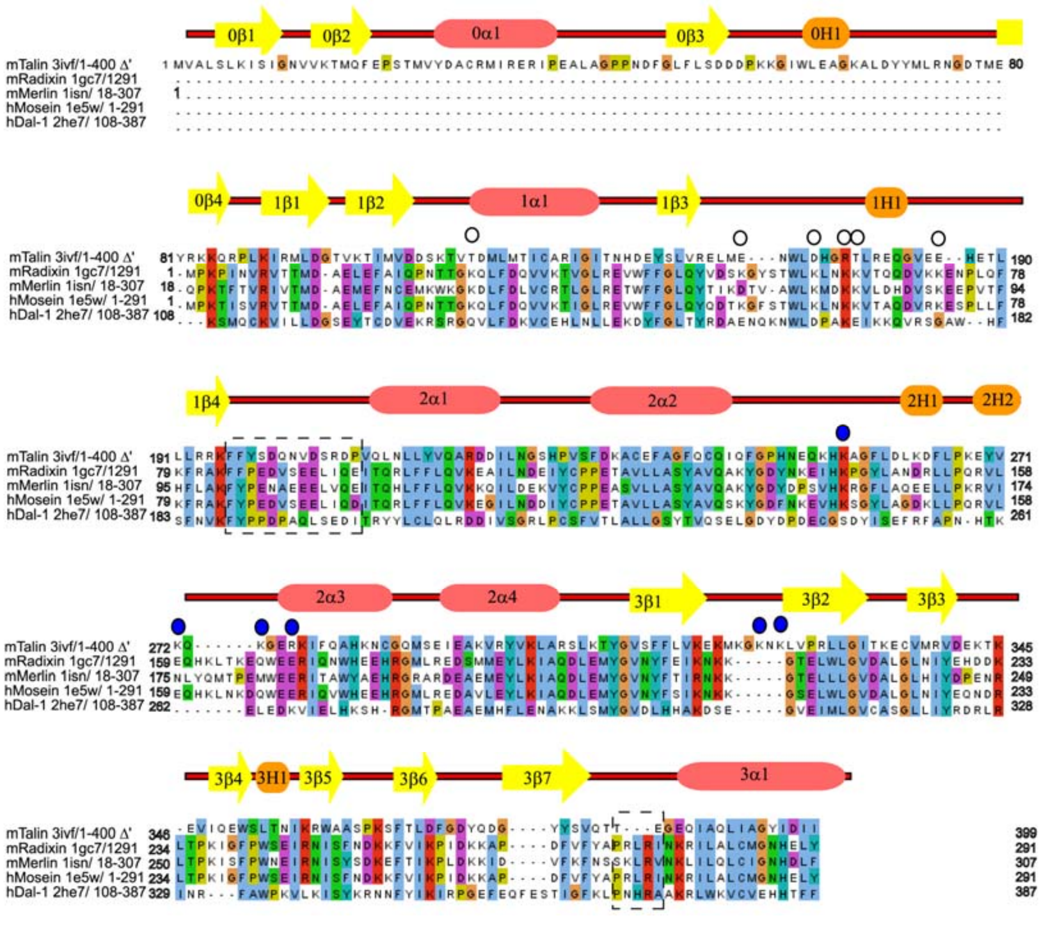

Figure S1, related to Figure 1.
Structure-based sequence alignment of the talin head fragment TH\` against the sequences of other FERM domains (top to bottom): talin, radixin (PDB ID 1GC7), moesin (1E5W), merlin (1ISN) and band 4.1-like protein (2HE7). Each FERM domain was aligned against talin F1-F3 using the DALI server and sequences manually adjusted using JalView. Similar residue types are identified by the colours using default JalView scheme. The secondary structure of talin is shown schematically above the sequence and all structural elements are annotated. The regions corresponding to F1-F2 linker and the N-terminal part of the helix 3α1 discussed in the text are marked by boxed areas. Talin and radixin residues involved in the interaction with membrane are indicated by filled and empty circles, respectively

Figure S2

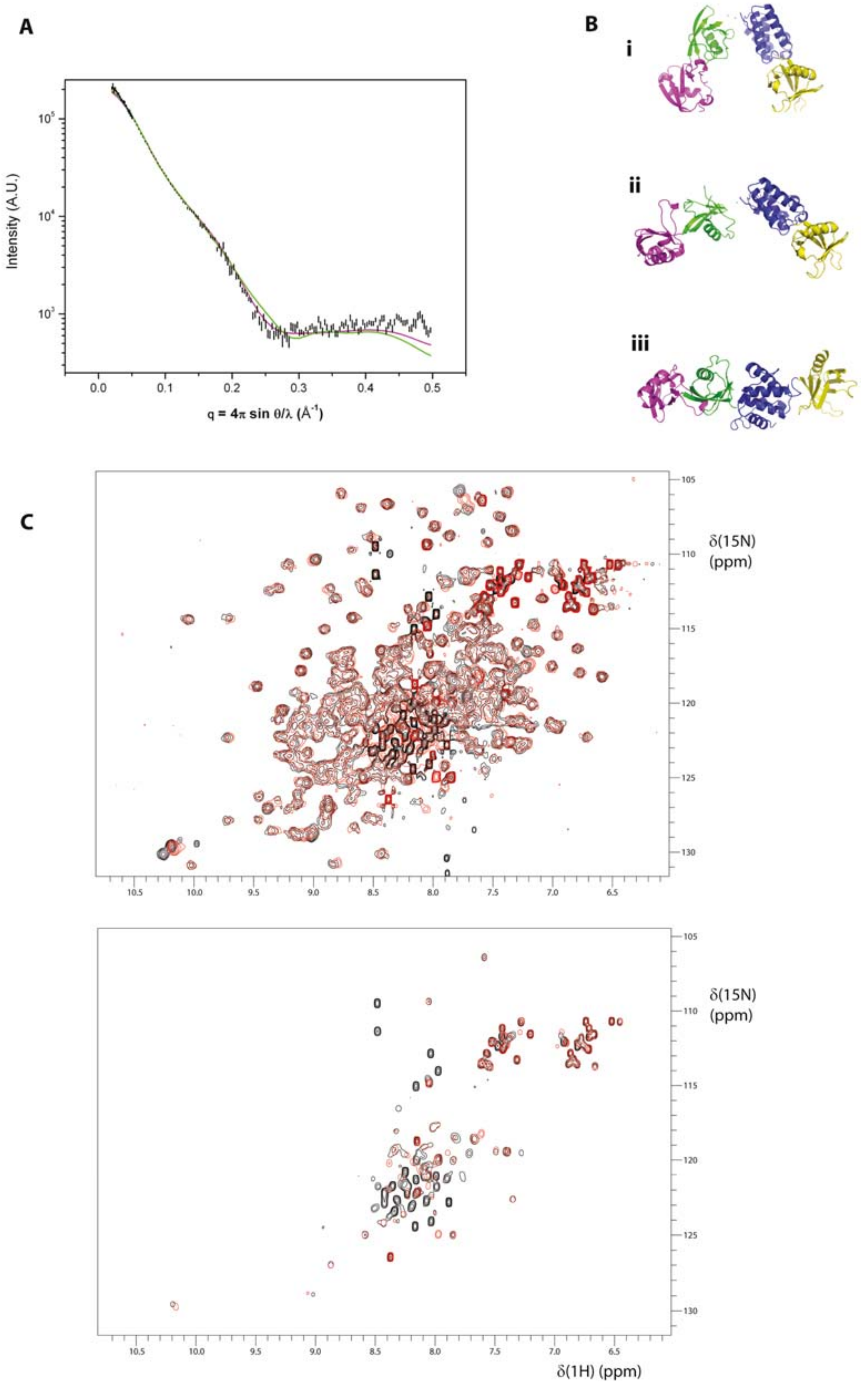

Figure S2, related to Figure 2. Solution analysis of the talin head. SAXS scattering profile reconstruction with Ensemble Optimised Method (EOM)

(**A**) Comparison between the experimental scattering profile (black) and profiles calculated from shape reconstruction with BUNCH (green) and the Ensemble Optimised Method (EOM) (red). The goodness of fit for GASBOR and EOM profiles versus experimental data is χ^2=3.2 and 2.5 respectively. In both methods the two double domains F0F1 and F2F3 were treated as independent rigid bodies. (**B**) Representatives of the three clusters of the EOM ensemble. The structures are

oriented to position the centers of all domains as close to the figure plane as possible. All the structures selected with EOM analysis have an open domain arrangement and can be grouped into three distinct clusters that differ by the angle between the double domains. One of the clusters corresponds to a nearly linear arrangement that is close to the crystal structure (population 18%), while the other two show more compact, but still open arrangements, with average angles of ~90° and ~70° (populations 47% and 35%, respectively). The domain orientation in the BUNCH model is intermediate between the fully open and partially closed EOM clusters. (**C**) Superposition of [^{1}H,^{15}N]-HSQC spectra of TH' (red) and the full talin head (residues 1-405, black). For the top panel contour levels were set close to the noise level to show broad low-intensity peaks corresponding to the folded regions on the proteins. The positions of the majority of the cross-peaks are the same for both proteins, with only small chemical shift changes detected for some of the cross-peaks. The similarity of the spectra demonstrates that the F1 loop removal does not affect the structure of the talin head. The bottom panel corresponds to high contour level to highlight most intense peaks of the spectra corresponding to the dynamic unstructured regions. The majority of such peaks present in the spectrum of the full talin head corresponds to the F1 linker as they disappear in the spectra of TH' upon the linker removal. The high intensity of the peaks and low dispersion of their ^{1}H chemical shifts evidences that the F1 linker in the full talin head is unstructured and dynamic. The NMR spectra were measured for 0.2 mM talin samples in 20mM sodium phosphate, 50mM NaCl, 2mM DTT, pH 6.5 buffer using Bruker Avance II 600 MHz spectrometer equipped with a CryoProbe. Proteins were expressed using 2xM9 minimal medium containing 1 g/L $^{15}NH_4Cl$.

Figure S3

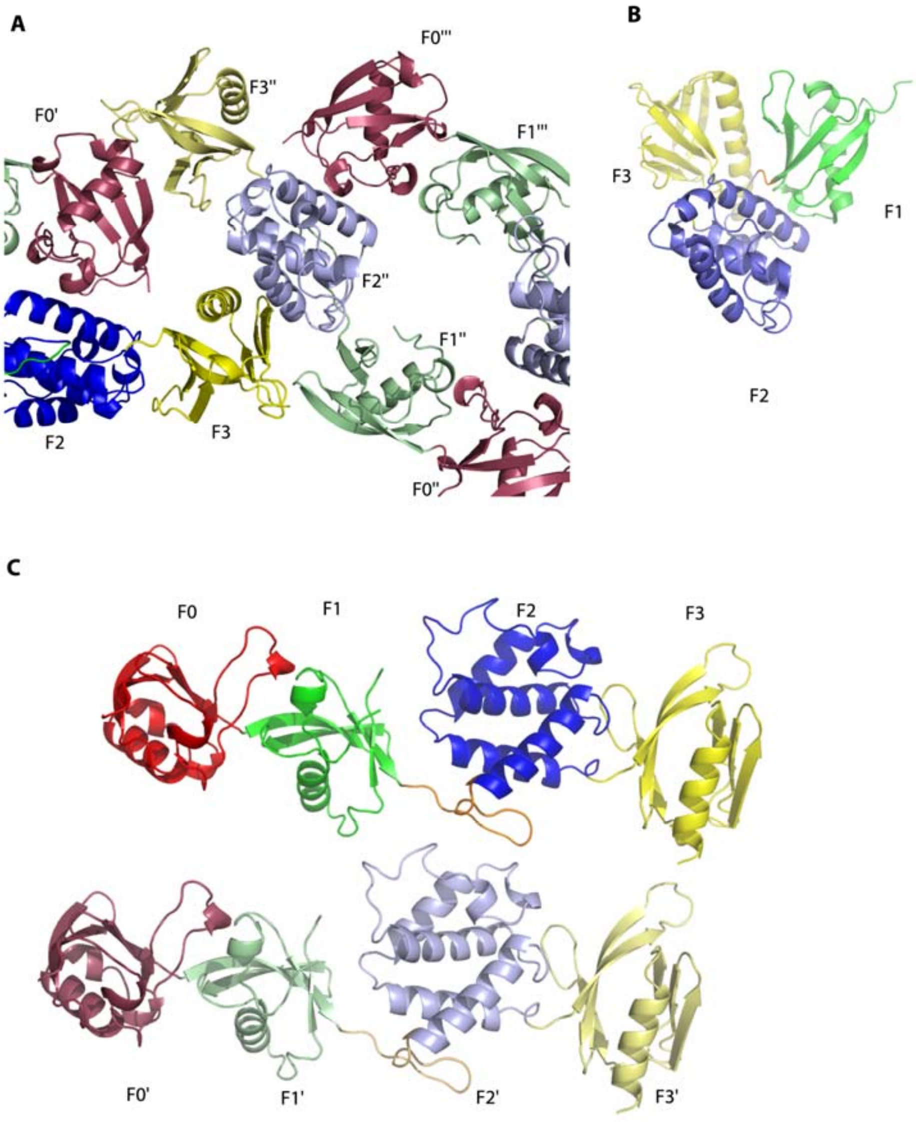

Figure S3, related to Figure 3. Crystal packing of the talin TH’ fragment
(**A**) Proximity of F1, F2 and F3 domains in the crystal form. Four molecules are shown identified as F0-F3, F0-F3’, F0-F3’’ and F0-F3’’’. In the arrangement that puts F1, F2 and F3 close to each other the F1F2’’ double domain of one molecule is contacting F3 domain of another molecule. Note that the relative domain positions are completely different to the canonical FERM clover-leaf structure, where F1 and F3 are on opposite sides of F2 (B). In addition, the contacts between the domains are limited and non-specific. (**B**) Domain arrangement in radixin FERM fragment that has the same F2 orientation as F2’’ of talin in (A). (**C**) Crystal packing of TH’ illustrating limited crystal contacts of the F1-F2 loop region (orange).

Figure S4

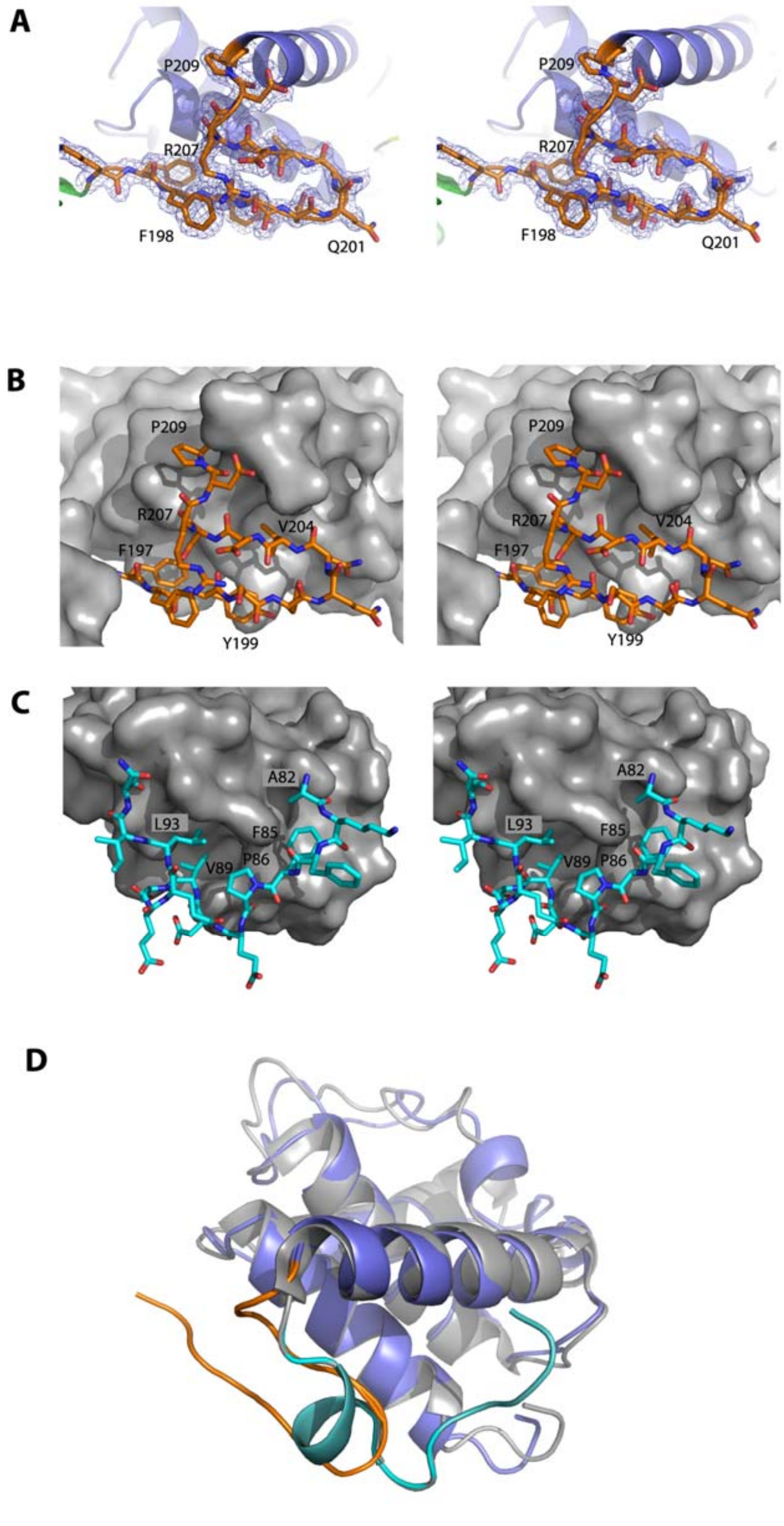

Figure S4, related to Figure 4. The F1-F2 linker regions makes has distinctly different packing against the F2 domain in talin and radixin

(A) Stereo view of the electron density from a 2Fo-Fc map corresponding to residues A195 through to P209, contoured at 0.8 σ, superimposed on the talin structure. For clarity of representation the R207 side chain has been omitted. (B) Stereo view of the docking of the β-hairpin formed in the F1-F2 linker region of talin (stick representation) against the surface of the F2 domain (grey). Residues

stabilizing the hairpin packing are marked. (C) Stereo view of the docking of the F1-F2 linker region of radixin (PDB ID 1GC7, stick representation) against the surface of the F2 domain (grey). Residues stabilizing the packing are marked. Note that the orientation of the F2 domain differs between (B) and (C) to optimize the display of the linker region. (D) Superposition of the F2 domain of talin and radixin in the orientation corresponding to (C).

SUPPLEMENTAL EXPERIMENTAL PROCEDURES

Small angle X-ray scattering

SAXS experiments were carried out at station 2.1 of the Synchrotron Radiation Source at Daresbury using a multiwire gas detector covering a momentum transfer range of $0.02 Å^{-1} < q < 0.70 Å^{-1}$ ($q=4\pi \sin \theta/\lambda$ where 2θ is the scattering angle and λ the X-ray wavelength of 1.54 Å). Measurements on TH' were performed at 4°C at concentrations of 2 and 10 mg/ml in 20mM sodium phosphate, 50mM NaCl, 2mM DTT, pH 6.5. Data were accumulated in 60-s frames and before averaging frames were inspected for X-ray-induced damage or aggregation. No protein aggregation was detected and the linearity of the Guinier plot indicates that the protein solutions were homogeneous. The background was subtracted using the scattering from the buffer solution alone. Data reduction was carried out with software provided at SRS Daresbury, and subsequent analysis was carried out with programs from the ATSAS package (Konarev et al., 2006). Scattering profiles were calculated from the crystal structure using CRYSOL (Svergun et al., 1995). A*b initio* bead modeling was performed with the program GASBOR (Svergun et al., 2001) which represents the protein as a chain of dummy residues centered at the Cα positions. Nine independent GASBOR runs were averaged using DAMAVER and SUBCOMB (Konarev et al., 2006) to obtain a representative molecular shape. Rigid body single model reconstruction was conducted with the program BUNCH (Petoukhov and Svergun, 2005). The double domains F0F1 and F2F3 were treated as two independent rigid bodies in conformations corresponding to those in the crystal structure of TH′. The relative orientation of the double domains was optimized to achieve the best agreement with the experimental scattering profile. For EOM reconstruction (Bernado et al., 2007) a pool of 10,000 random conformers was generated from the crystal structure by moving the F0F1 double domain relative to the F2F3 double domain around the flexible hinge defined by residues R195-K196. The scattering curve was calculated for each of these conformers, and a genetic algorithm was used to find the optimum combination of these scattering curves to account for the experimental data. Standard parameters were used in the calculations.

Phospholipid Cosedimentation Assay

Dried phospholipid films were swollen at 5mg/ml in 20mM Hepes (pH 7.4), 0.2mM EGTA for 3h at 42°C. The vesicles were then centrifuged (20,000g for 20min at 4°C) and the pellet was resuspended in the same buffer at 5mg/ml. Protein samples were diluted into 20mM Tris/HCl, 0.1mM EDTA, 15mM mercaptoethanol, pH 7.4. After centrifugation proteins (0.15mg/ml) were incubated (30min, 25°C) in the absence or presence of phospholipid vesicles (0.5mg/ml; 200µl total volume) followed by centrifugation (25,000g for 20min at 4°C). Pellet and supernatant fractions were analyzed by SDS-PAGE and proteins detected by Coomassie-blue staining.

SUPPLEMENTAL REFERENCES

Bernado, P., Mylonas, E., Petoukhov, M.V., Blackledge, M., and Svergun, D.I. (2007). Structural Characterization of Flexible Proteins Using Small-Angle X-ray Scattering. J. Am. Chem. Soc. *129*, 5656-5664.

Konarev, P.V., Petoukhov, M.V., Volkov, V.V., and Svergun, D.I. (2006). ATSAS 2.1, a program package for small-angle scattering data analysis. J. Appl. Cryst. *39*, 277-286.

Petoukhov, M.V., and Svergun, D.I. (2005). Global Rigid Body Modeling of Macromolecular Complexes against Small-Angle Scattering Data. Biophys. J. *89*, 1237-1250.

Svergun, D., Barberato, C., and Koch, M.H.J. (1995). CRYSOL -a program to evaluate X-ray solution scattering of biological macromolecules from atomic coordinates. J. Appl. Cryst. *28*, 768-773.

Svergun, D.I., Petoukhov, M.V., and Koch, M.H.J. (2001). Determination of Domain Structure of Proteins from X-Ray Solution Scattering. J. Appl. Cryst. *80*, 2946-2953.
